# CCN2/CTGF—A Modulator of the Optic Nerve Head Astrocyte

**DOI:** 10.3389/fcell.2022.864433

**Published:** 2022-04-14

**Authors:** Andrea E. Dillinger, Gregor R. Weber, Matthias Mayer, Magdalena Schneider, Corinna Göppner, Andreas Ohlmann, Mikhail Shamonin, Gareth J. Monkman, Rudolf Fuchshofer

**Affiliations:** ^1^ Institute of Human Anatomy and Embryology, University of Regensburg, Regensburg, Germany; ^2^ Faculty of Electrical Engineering and Information Technology, Ostbayerische Technische Hochschule Regensburg, Regensburg, Germany; ^3^ Department of Ophthalmology, University Hospital, Ludwig-Maximilians-University Munich, Munich, Germany

**Keywords:** glaucoma, optic nerve, growth factors, astrocytes, stiffness, extracellar matrix, glial lamina

## Abstract

In primary open-angle glaucoma (POAG), a neurodegenerative disease of the optic nerve (ON) and leading cause of blindness, the optic nerve head (ONH) undergoes marked structural extracellular matrix (ECM) changes, which contribute to its permanent deformation and to degeneration of ON axons. The remodeling process of the ECM causes changes in the biomechanical properties of the ONH and the peripapillary sclera, which is accompanied by an increased reactivity of the resident astrocytes. The molecular factors involved in the remodeling process belong to the Transforming growth factor (TGF)-β superfamily, especially TGF-β2. In previous publications we showed that TGF-β2 induced ECM alterations are mediated by Cellular Communication Network Factor (CCN)2/Connective Tissue Growth Factor (CTGF) and recently we showed that CCN2/CTGF is expressed by astrocytes of the ON under normal conditions. In this study we wanted to get a better understanding of the function of CCN2/CTGF under normal and pathologic conditions. To this end, we analyzed the glial lamina and peripapillary sclera of CCN2/CTGF overexpressing mice and studied the effect of CCN2/CTGF and increasing substratum stiffness on murine ON astrocytes *in vitro*. We observed enhanced astrocyte reactivity in the ONH, increased ECM protein synthesis in the peripapillary sclera and increased *Ccn2/Ctgf* expression in the ONH during the pathologic development *in situ*. CCN2/CTGF treatment of primary murine ON astrocytes induced a higher migration rate, and increase of ECM proteins including fibronectin, elastin and collagen type III. Furthermore, the astrocytes responded to stiffer substratum with increased glial fibrillary acidic protein, vimentin, actin and CCN2/CTGF synthesis. Finally, we observed the reinforced appearance of CCN2/CTGF in the lamina cribrosa of glaucomatous patients. We conclude that reactive changes in ONH astrocytes, induced by the altered biomechanical characteristics of the region, give rise to a self-amplifying process that includes increased TGF-β2/CCN2/CTGF signaling and leads to the synthesis of ECM molecules and cytoskeleton proteins, a process that in turn augments the stiffness at the ONH. Such a scenario may finally result in a vicious circle in the pathogenesis of POAG. The transgenic CTGF-overexpressing mouse model might be an optimal model to study the chronic pathological POAG changes in the ONH.

## Introduction

Primary open angle glaucoma (POAG), a neurodegenerative disease of the optic nerve (ON), is one of the leading cause of blindness in the western hemisphere (Quigley, 1996; Resnikoff et al., 2004). In course of the disease, ON axons become damaged at the optic nerve head (ONH), leading to progressive loss of retinal ganglion cells (RGCs) (Quigley, 2011), which is characterized by an excavation or cupping of the optic disk, a key clinical feature of glaucoma differentiating it from other optic neuropathies (Quigley et al., 1983). Multiple, randomized clinical studies revealed that intraocular pressure (IOP) is a major risk factor for the axon loss in POAG and that lowering IOP reduces its progression (Anderson, 1998a; Anderson, 1998b) (Anderson, 1998b; Kass et al., 2002). The location of glaucomatous damage is at the scleral opening where RGC axons leave the eye (Quigley et al., 1983). The axons are supported at the passage by a mesh-like structure, which constitutes of connective tissue and/or cellular elements depending on the species (Anderson, 1969; Radius and Gonzales, 1981; Quigley, 1987; May and Lutjen-Drecoll, 2002; Howell et al., 2007; Sun et al., 2009). In humans, the lamina cribrosa is a sieve-like plate of connective tissue and elastic fibers, lined by a dense meshwork of astrocytes (Quigley, 2011). During the pathogenesis of POAG the lamina cribrosa undergoes structural changes. The connective tissue organization is markedly altered and the sheaths around the capillaries are significantly thickened in the prelaminar region of the ONH (Pena et al., 1998; Pena et al., 2001; Gottanka et al., 2005; Tektas et al., 2010). The process includes alterations in quality and quantity of ECM components, which causes changes in the biomechanical properties of the affected tissue (Hopkins et al., 2020). Also, cellular changes in the resident astrocytes of the ONH are observed during the pathogenesis of the POAG, indicated by an increased reactivity, which is characterized by morphologic changes and variations in their expression patterns (Pena et al., 1999; Hernandez, 2000; Hernandez et al., 2002). While IOP is thought to be an important factor in the process of lamina cribrosa deformation, the causative mechanisms of axonal degeneration are not completely understood, but likely include neurotrophic deprivation due to axonal compression, lamina cribrosa deformation and finally the disruption of axonal transport.

In the last decade many different mouse glaucoma models were generated to achieve a better understanding of the pathological alterations during the progression of the disease, even though mice do not have a collagenous lamina cribrosa (Johnson and Tomarev, 2010). In mice, a honeycomb structure is built up by astrocytes, forming a glial lamina, where the ON axons pass through and leave the eye (May and Lutjen-Drecoll, 2002; Howell et al., 2007; Sun et al., 2009). In mice and humans, the ON axons are lined by astrocytes in this region and in mouse or rat glaucoma models the astrocytes show similar alterations of reactivity after IOP elevation like glaucomatous patients (Radius and Gonzales, 1981; Elkington et al., 1990; Ye and Hernandez, 1995; Trivino et al., 1996; Oyama et al., 2006; Sun et al., 2009; Tehrani et al., 2016; Sun et al., 2017; Wang et al., 2017).

The molecular mechanisms contributing to the disease are not entirely known yet, but increased amounts of Transforming growth factor (TGF) -β2 were detected in the ONH of POAG patients (Pena et al., 1999; Zode et al., 2011). There is evidence that reactive astrocytes are the major source of growth factor and ECM component upregulation, as it was shown that TGF-β2 is extensively localized to reactive astrocytes in the glaucomatous ONH (Pena et al., 2001). *In vitro* studies could show that TGF-β2 leads to an increased synthesis of ECM proteins in human ONH astrocytes, and that this effect is dependent on its downstream mediator Cellular Communication Network Factor/Connective Tissue Growth Factor (CCN2/CTGF) (Fuchshofer et al., 2005). CCN2/CTGF is a member of the CCN family of matricellular regulatory proteins and is involved in fibrotic processes in many different diseases (Leask et al., 2002; Brigstock, 2003; Rachfal and Brigstock, 2005). In the healthy human eye CCN2/CTGF is described to be present in many different compartments and it shows high expression especially in the trabecular meshwork (TM) (Tomarev et al., 2003; van Setten et al., 2016). Moreover, glaucomatous Schlemm’s canal cells showed a significantly higher CCN2/CTGF expression than healthy controls (Overby et al., 2014). In the aqueous humor CCN2/CTGF is a general component, furthermore CCN2/CTGF levels are increased in patients with pseudoexfoliation glaucoma (van Setten et al., 2002; Browne et al., 2011). Finally, a direct implication of CCN2/CTGF in the dysregulation of the aqueous humor outflow facility was demonstrated in transgenic animals with a lens specific expression of CCN2/CTGF (Junglas et al., 2012). In this mouse model, the chicken βB1-crystallin promotor was used to direct high and specific expression of transgenes to lens fibers of the mouse eye (Duncan et al., 1996; Junglas et al., 2012). In the lenses of βB1-CTGF1 mice, but not in the rest of the eye or in the lenses or eyes of wild-type littermates a strong and specific expression of CCN2/CTGF mRNA was detected. Furthermore, this transgenic expression led to the secretion of high amounts of CCN2/CTGF into the aqueous humor of the transgenic animals. The lens specific expression of CCN2/CTGF causes an increased IOP and decline in number of ON axons. Both effects continue to increase with increasing age (Junglas et al., 2012). Recently we could show that astrocytes are the cellular source of CCN2/CTGF in the murine ON and ONH (Dillinger et al., 2021). A detailed analysis of the CCN2/CTGF distribution in the ONH of glaucomatous eyes is missing.

In this study we analyzed the astrocytic reactivity and CCN2/CTGF level in the ON and ONH and the ECM protein synthesis in the peripapillary sclera in the βB1-CTGF1 murine glaucoma model. Furthermore, we investigated the CCN2/CTGF distribution in the ONH in healthy and glaucomatous human eyes. We analyzed *in vitro* the direct effect of CCN2/CTGF on ECM components, like fibronectin or collagen and the cytoskeleton in primary murine ON astrocytes. Finally, we investigated whether cultured primary murine ON astrocytes can sense even small changes in stiffness in their surrounding matrix.

## Results

### Increased Astrocyte Reactivity in the Glial Lamina of a Murine Glaucoma Model

**FIGURE 1:**
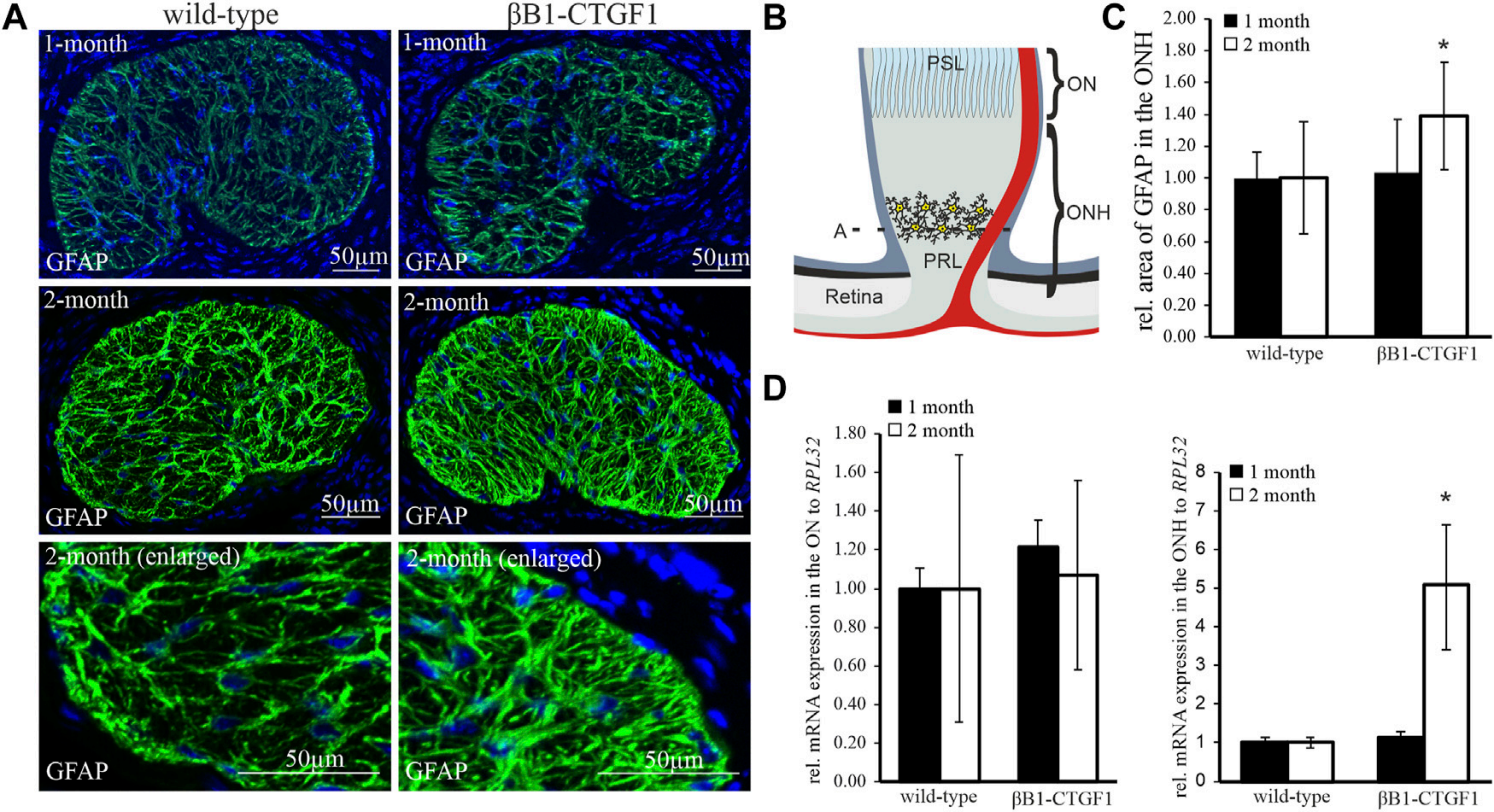
GFAP expression and synthesis in the ON and ONH in a murine glaucoma model. **(A)** GFAP immunoreactivity in the glial lamina of 1-month (green, upper panel) and 2 months (green, middle and lower panel) old βB1-CTGF1 mice compared to WT controls. Immunoreactivity of GFAP was not altered in 1-month old TG and WT animals (green, upper panel). 2 month old βB1-CTGF1 mice showed an increased GFAP immunoreactivity compared to WT control (green, middle panel). Lower panel shows a magnified detail of the middle panel. Nuclei are stained with Dapi (blue). **(B)** Schmatic illustration of the optic nerve. A depicts the region in the glial lamina where cross sections were obtained. ONH (unmyelinated part) and ON (myelinated part) were used for molecular analysis. **(C)** Quantification of immunohistochemical staining of GFAP in the glial lamina is not altered in 1 month old TG and WT animals (WT: *n* = 5; TG: *n* = 4). GFAP immunoreactivity is increased in 2 month old TG animals compared to WT littermates (WT: *n* = 10, TG: *n* = 13; *p = 0.039; two-tailed *t*-test compared to theoretical mean of 1 (normalized control)). Mean value of WT animals (control) was set at 1. **(D)** RT-PCR analyses revealed no alteration in the *Gfap* mRNA expression in the ON of 1-month (WT: *n* = 7; TG: *n* = 5) and 2 month old βB1-CTGF1 mice (WT: *n* = 15; TG: *n* = 16) compared to WT controls. In the ONH the *Gfap* mRNA expression is increased in the 2 month old TG compared to WT animals (WT: *n* = 6, TG: *n* = 5; *p = 0.04). The mRNA expression of *Gfap* is not altered in 1 month old animals (WT: *n* = 6, TG: *n* = 5). Mean value of WT animals (control) was set at 1. *RPL32* was used to normalize mRNA expression. Data represented as mean ± SEM. PRL, Prelaminar region; PSL, Postlaminar region; ON, optic nerve; ONH, optic nerve head.

An increased reactivity of resident astrocytes in POAG is indicated by morphologic changes and changes in the expression pattern of Glial fibrillary acidic protein (GFAP), a specific marker for astrocytes. Therefore, we investigated the distribution of GFAP in tangential sections of the glial lamina of 1 month and 2 month old βB1-CTGF1 mice, to analyze astrocyte morphology and expression profile. 1 month-old βB1-CTGF1 mice showed no changes in IOP compared to their wildtype (WT) littermates (WT: 14.71 ± 1.68; TG: 15.11 ± 3.34; *p* = 0.75). In contrast, 2 month-old transgenic (TG) mice showed a significant increase in IOP compared to WT controls (WT: 15.34 ± 2.08; TG: 16.70 ± 1.47; *p* = 0.018). GFAP immunostaining in the glial lamina of 1 month old TG and WT shows no alteration either in the GFAP synthesis or astrocyte morphology (Figure 1A, upper panel). In 2 month-old TG animals, the immunoreactivity for GFAP was increased in the glia lamina, compared to WT littermates (Figure 1A, middle and lower panel). The morphological analysis of the glial lamina showed a thickening of the astrocytic processes and the open spaces between the astrocyte processes were diminished in the TG animals in comparison to WT controls. The schematic illustration in Figure 1B shows the region where cross sections of the glial lamina and ON and ONH samples for molecular analysis were obtained. Quantification of the GFAP stained area in the glial lamina showed a significant increase in the TG animals compared to WT littermates in 2 month-old animals, but not in 1 month-old mice (1 month-old: WT: 1 ± 0.16; TG: 1.03 ± 0.33; 2 month-old: WT: 1.00 ± 0.35; TG: 1.39 ± 0.34; Figure 1C). Real-time RT-PCR analyses were performed to determine the expression level of *Gfap* in the ONH, containing the unmyelinated part of the nerve, and in the ON, comprised of the remaining myelinated nerve of 1 month and 2 month old βB1-CTGF1 and WT mice (Figure 1B). The *Gfap* mRNA expression analyses of the 1 month old TG mice shows no changes either in the ON (WT: 1 ± 0.06; TG: 1.19 ± 0.24) or the ONH (WT: 1 ± 0.14; TG: 1.14 ± 0.15) in comparison to their WT littermates (Figure 1D). Interestingly, real-time RT-PCR experiments showed a dramatic increase of *Gfap* in the ONH of 2 month old TG animals, which have a significantly enhanced intraocular pressure compared to WT controls (Junglas et al., 2012) (WT: 1 ± 0.13; TG: 5.08 ± 1.62; Figure 1C). Intriguingly, the mRNA analyses of the ON show no change in the *Gfap* expression (WT: 1 ± 0.68; TG: 0.85 ± 0.45; Figure 1D).

### Fibronectin and the Amount of Filamentous Actin are Increased in the Optic Nerve Head of 2 Month old Transgenic βB1-Connective Tissue Growth Factor1 Mice

**FIGURE 2:**
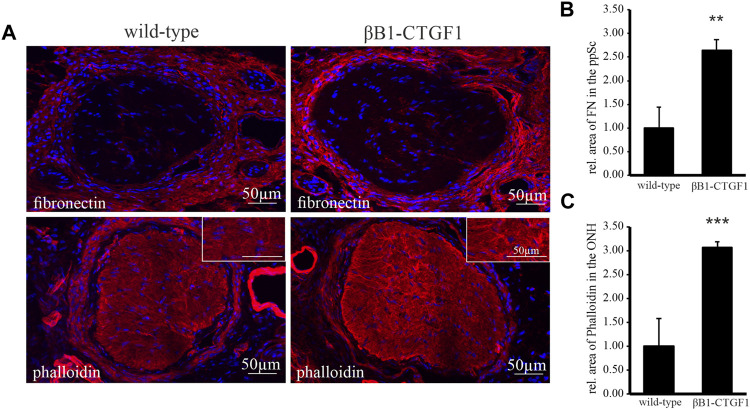
Immunoreactivity of fibronectin and labeling of filamentous actin in the ON and ONH in a murine glaucoma model. **(A)** Immunoreactivity of fibronectin (red, upper panel) is increased in the peripapillary sclera of βB1-CTGF1 mice compared to WT controls. Filamentous actin labeled by Phalloidin (red, lower panel) in increased in the ON of βB1-CTGF1 mice compared to WT controls. Nuclei are stained with Dapi (blue). **(B)** Quantification of immunohistochemical staining of fibronectin show a significant increase in the peripapillary sclera of TG animals compared to controls (TG: *n* = 4; WT: *n* = 5; **p = 0.002). **(C)** Quantification of immunohistochemical staining of phalloidin labeled filamentous actin show a significant increase in the ON of TG animals compared to controls (TG: *n* = 5; WT: *n* = 5; ***p = 0.0001).

It is suggested that axonal damage is associated with alterations of the biomechanical properties of the peripapillary sclera (Coudrillier et al., 2012), which translates the IOP changes to the glial lamina and ON axons (Kimball et al., 2014). As the 2 month old TG βB1-CTGF1 mice show intense changes in astrocytes morphology in the ONH (Figure 1A), we focused on the 2 month old animals for the analysis of fibronectin and filamentous actin in the peripapillary sclera (Figure 2A). The immunohistochemical analysis of fibronectin and its distribution in tangential sections of the glial lamina region showed a marked increase of fibronectin in the peripapillary sclera in TG in comparison to controls. The localization of fibronectin within the glial lamina was mostly around the blood vessels. More interestingly the intensity of the staining was enhanced in TG animals compared to WT mice (Figure 2A, upper panel). The quantification of fibronectin stained areas showed a significant increase in the peripapillary sclera region of 2 month old TG mice compared to WT littermates (WT: 1 ± 0.45; TG, 2.64 ± 0.24; Figure 2B). The observed changes were accompanied by a considerable increase in the amount of filamentous actin in the ON of βB1-CTGF1 compared to their WT littermates. The increase in phalloidin labeled actin was especially pronounced next to the peripapillary sclera (Figure 2A, lower panel). The measurement of the area of phalloidin labeled filamentous actin in the ONH could show an increase in the TG animals in comparison to WT littermates (WT: 1 ± 0.58; TG: 3.07 ± 0.11; Figure 2C).

### Cellular Communication Network Factor 2/Connective Tissue Growth Factor is Increased in a Murine Glaucoma Model

**FIGURE 3:**
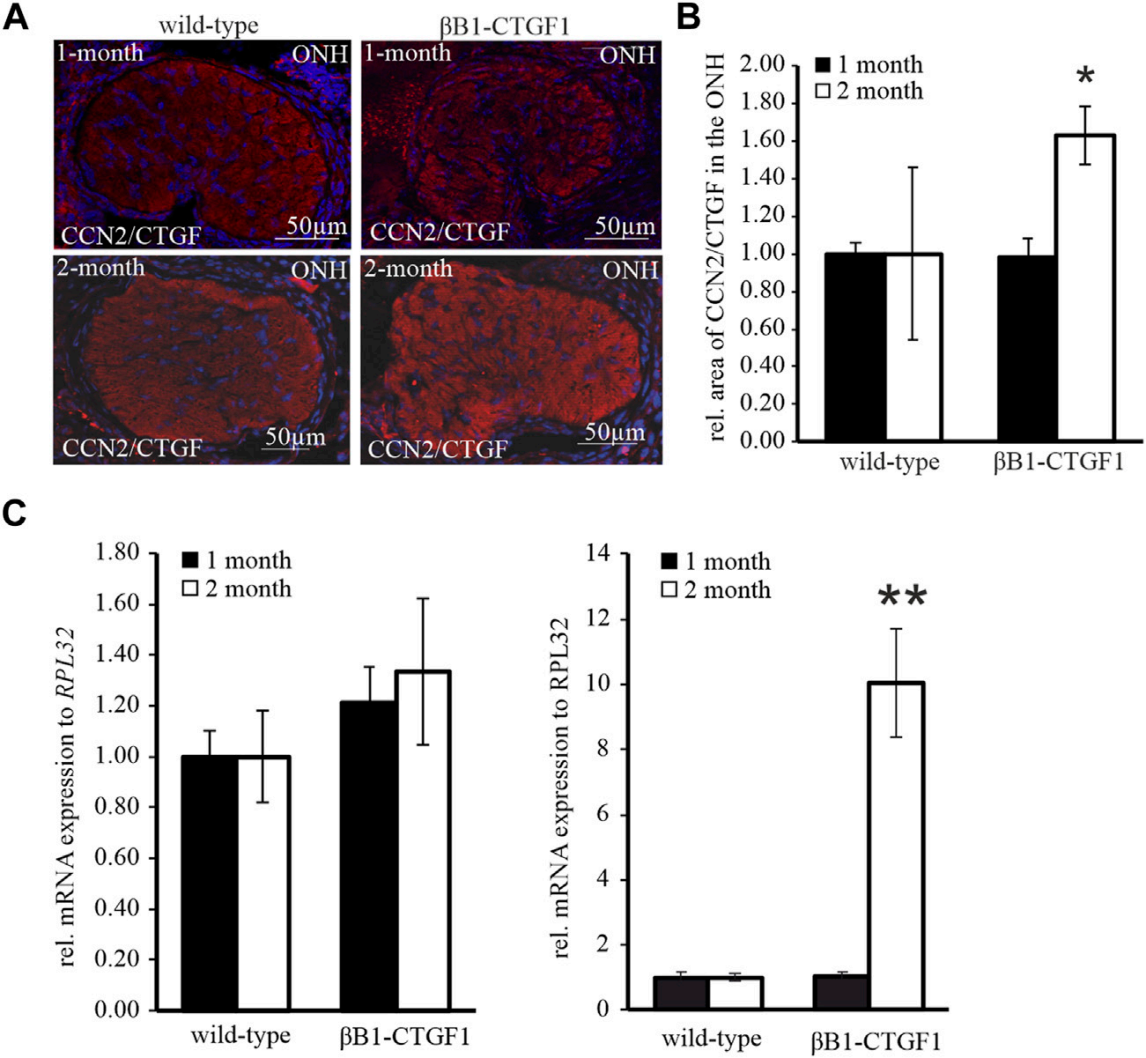
CCN2/CTGF expression and protein synthesis in the ON and ONH in a murine glaucoma model. **(A)** CCN2/CTGF immunoreactivity (red) in the glial lamina of 1 month (upper panel) and 2 months (lower panel) old βB1-CTGF1 mice compared to WT controls. Immunoreactivity of CCN2/CTGF was not altered in 1-month old TG and WT animals (upper panel). 2 month old βB1-CTGF1 mice showed an increased CCN2/CTGF immunoreactivity compared to WT control (middle panel). Nuclei are stained with Dapi (blue). **(B)** Quantification of immunohistochemical staining of CCN2/CTGF in the glial lamina is not altered in 1-month old TG and WT animals (WT: *n* = 5; TG: *n* = 5). CCN2/CTGF immunoreactivity is increased in 2 month old TG animals compared to WT littermates (WT: *n* = 5; TG: *n* = 5; *p = 0.028; unpaired two-tailed *t*-test). Mean value of WT animals (control) was set at 1. **(C)** RT-PCR analyses revealed no alteration in the CCN2/CTGF mRNA expression in the ON of 1-month (WT: *n* = 7; TG: *n* = 4) and 2 month old βB1-CTGF1 mice (WT: *n* = 14; TG: *n* = 16) compared to WT controls. In the ONH the CCN2/CTGF mRNA expression is increased in the 2-month old TG compared to WT animals (WT: *n* = 6; TG: *n* = 5; **p = 0.0006). The mRNA expression of CCN2/CTGF is not altered in 1-month old animals (WT: *n* = 6; TG: *n* = 3). Mean value of WT animals (control) was set at 1. *RPL32* was used to normalize mRNA expression. Data represented as mean ± SEM. ONH, optic nerve head.

To address the question, whether CCN2/CTGF is altered related to increased astrocyte reactivity and enhanced IOP, the immunoreactivity of CCN2/CTGF in the glial lamina was analyzed. A faint staining throughout the entire ON tissue with no obvious preference for glial or neuronal tissue is observed in TG and WT mice (Figure 3A). Immunoreactivity against CCN2/CTGF shows no changes in the glial lamina of 1-month old TG mice, compared to WT littermates (Figure 3A). Furthermore, quantification of CCN2/CTGF stained area in the glial lamina sections could not identify any variation (WT: 1.00 ± 0.05; TG: 0.98 ± 0.10; Figure 3B). 2 month old WT mice showed the same distribution for CCN2/CTGF as observed for the 1 month old animals. Interestingly, the intensity of the immunoreactivity for CCN2/CTGF was increased in the 2 month old animals compared to WT animals (Figure 3A). Measurements of CCN2/CTGF stained area in the ONH show a significant increase in the TG mice in comparison to WT littermates (WT: 1.00 ± 0.46; TG: 1.63 ± 0.16; *p = 0.028; Figure 3B). mRNA analysis of *Ccn2/Ctgf* in the 1 month old animals showed similar results, which were seen for GFAP. *Ccn2/Ctgf* mRNA expression is not changed either in the ON (WT: 1 ± 0.10; TG: 1.21 ± 0.14) or in the ONH (WT: 1 ± 0.18; TG: 1.06 ± 0.14; Figure 3C) of 1 month old animals. Interestingly, real time RT-PCR experiments showed a huge increase of *Ccn2/Ctgf* in the ONH of TG animals compared to WT littermates (WT: 1 ± 0.12; TG: 10.04 ± 1.66; **p = 0.006; Figure 3C) in the 2 month old animals. Intriguingly, the mRNA analyses of the ON showed no change in the *Ccn2/Ctgf* expression (WT: 1 ± 0.18; TG: 1.33 ± 0.29; Figure 3C).

### Cellular Communication Network Factor 2/Connective Tissue Growth Factor is Increased in the Glaucomatous Optic Nerve

**FIGURE 4:**
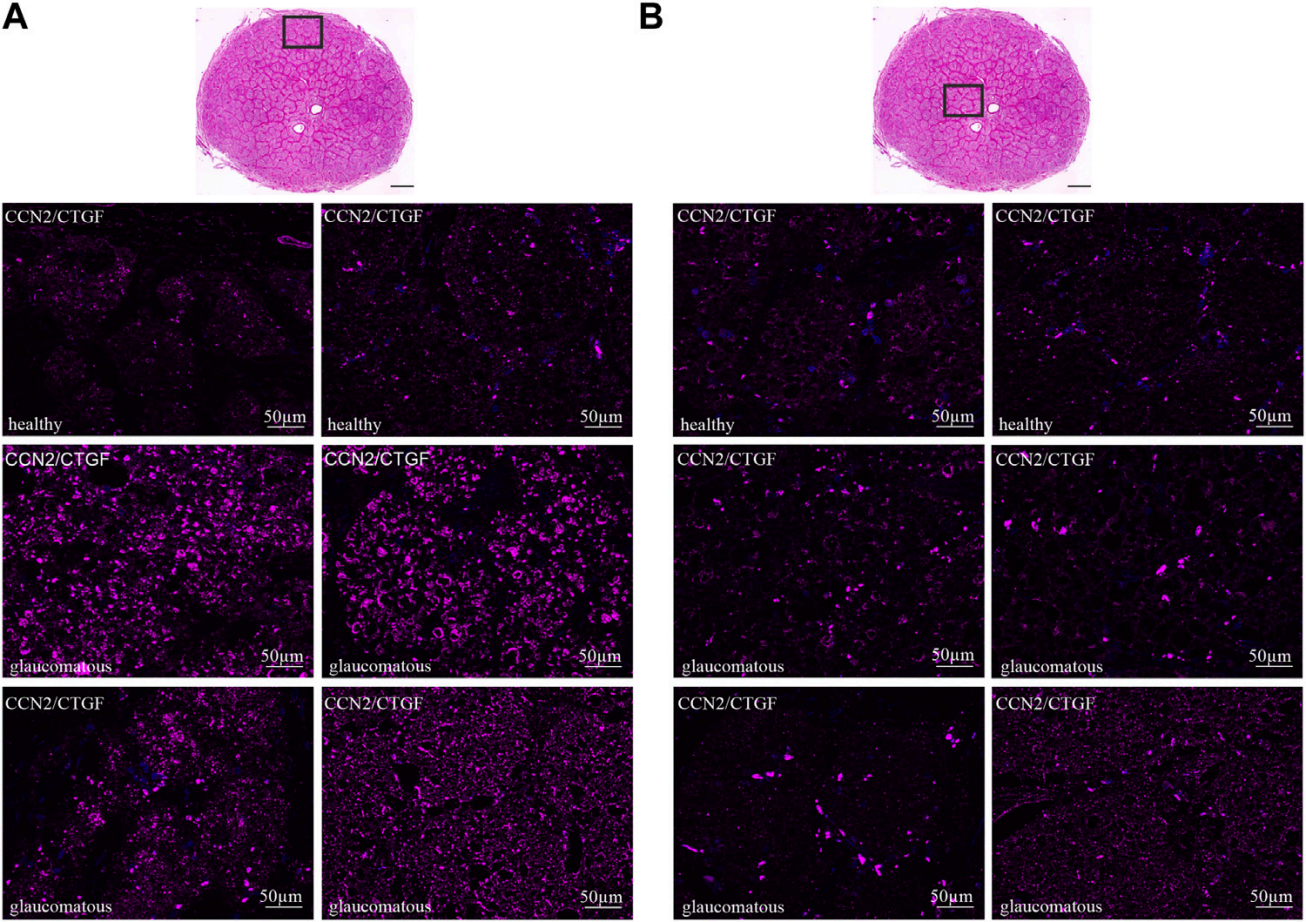
CCN2/CTGF in the healthy and glaucomatous ONH of the human eye. Tangential cross section with H&E staining in **A** and **B** represents the location of lower pictures within the ON indicated by the rectangle. **(A)** CTGF immunoreactivity (purple) in the border area of glaucomatous ON is increased compared to healthy control. **(B)** CTGF immunoreactivity is not altered in the central area of the glaucomatous ON compared to the healthy ON. Nuclei are stained with Dapi (blue).

We further investigated the CCN2/CTGF expression in the human ON under healthy and glaucomatous conditions. Therefore, we analyzed the CCN2/CTGF immunoreactivity in two healthy and four glaucomatous human ON (Figure 4). In the healthy human ON we found a weak, but consistent CCN2/CTGF signal in the entire ON, with no obvious preference for glial or neuronal tissue. Additionally, we could not reveal any difference of the CCN2/CTGF synthesis related to different regions of the ON (Figures 4A,B, upper panel). The border area (Figure 4A, upper panel) showed a similar CCN2/CTGF immunoreactivity as the central part of the healthy ON (Figure 4B, upper panel). To investigate whether an altered CCN2/CTGF synthesis can be observed under glaucomatous conditions, we analyzed the CCN2/CTGF immunoreactivity of four human glaucomatous ON in comparison to the healthy human ON. We could find an intense increase of CCN2/CTGF immunoreactivity in the border area of the glaucomatous ON compared to the healthy ON. This finding is consistent in all four analyzed glaucomatous ON (Figure 4A). In contrast we could not observe changes in the CCN2/CTGF intensity in the central area of the glaucomatous ON, except one of the glaucomatous ON which shows a slight increase (Figure 4B, lower panel, right ON).

### Transforming Growth Factor-β2 and Cellular Communication Network Factor2/Connective Tissue Growth Factor Induce Astrocyte Reactivity, Migration Rate and Alter the Cytoskeleton

**FIGURE 5:**
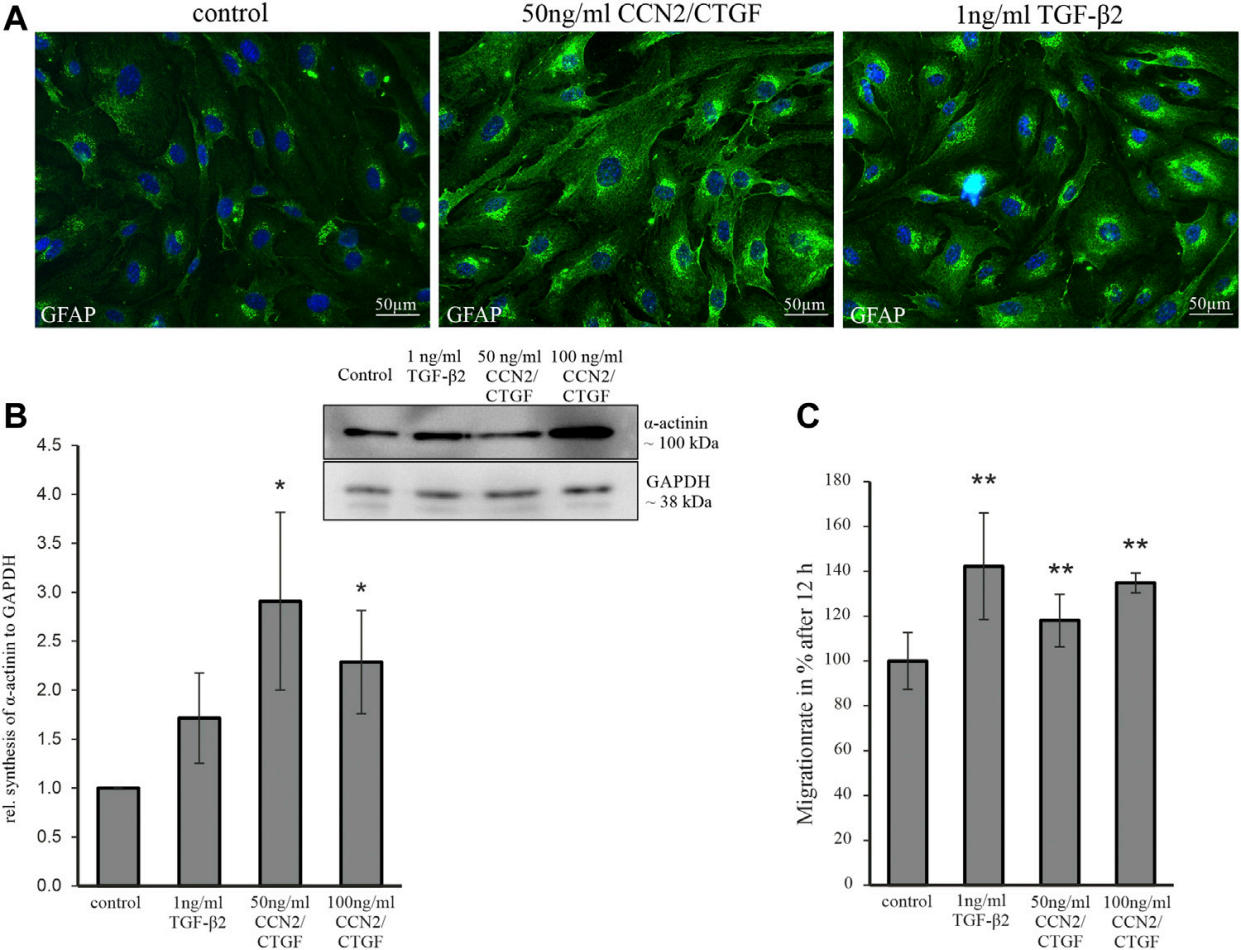
Analysis of astrocyte reactivity, the cytoskeleton and migration rate following treatment with TGF-β2 and CCN2/CTGF. **(A)** GFAP immunoreactivity (green) is increased in murine ON astrocytes following TGF-β2 and CCN2/CTGF treatment. Nuclei are stained with Dapi (blue) **(B)** Western Blot experiments analyses of α-actinin protein synthesis in murine ON astrocytes after the treatment with 1 ng/ml TGF-β2, 50 ng/ml CCN2/CTGF and 100 ng/ml CCN2/CTGF for 24 h. Densitometric analyses of Western Blot analyses shows the significant increase in α-actinin protein synthesis. Mean value of untreated cells (control) was set at 1. GAPDH was used to normalize protein intensity (*n* = 4; 50 ng CCN2/CTGF: *p = 0.029; 100 ng CCN2/CTGF: *p = 0.015; unpaired two-tailed *t*-test). **(C)** Scratch assay analyses show a significant increased migration rate for murine ON astrocytes after the treatment with ng/ml TGF-β2, 50 ng/ml CCN2/CTGF and 100 ng/ml CCN2/CTGF for 12 h. Mean value of migration rate of control cells was set to 100% (control: n = 15; TGF-β2: *n* = 12, **p = 0.008; 50 ng/ml CCN2/CTGF: *n* = 10, *p = 0.001; 100 ng/ml CCN2/CTGF: *n* = 4, **p = 0.0001). Data represented as mean ± SD.

Since TGF-β2 induces a reactive phenotype in human ONH astrocytes *in vitro* and promotes the synthesis of GFAP and ECM proteins (Fuchshofer et al., 2005; Kirschner et al., 2021), we wanted to know, whether cultured murine ON astrocytes react in the same manner. The TGF-β2 induced ECM synthesis is dependent on CCN2/CTGF (Fuchshofer et al., 2005), so we wanted to investigate the direct effects of CCN2/CTGF in murine ON astrocytes *in vitro*. For this reason, we established a murine ON astrocyte cell culture (see Supplementary Methods S1; Supplementary Figure S1). Astrocytes were treated either with 1 ng/ml TGF-β2, 50 ng/ml and 100 ng/ml CCN2/CTGF and were compared to untreated control cells. Treatment with 1 ng/ml TGF-β2 led to marked increase in GFAP immunoreactivity in murine ON astrocytes in comparison to untreated control cells. Treatment with 50 ng/ml CCN2/CTGF caused a comparable reaction to that seen after TGF-β2 treatment (Figure 5A). The evaluation of the effect of both growth factors on the actin cytoskeleton showed a significant increase in α-actinin protein synthesis after CCN2/CTGF treatment, which is necessary for actin filament crosslinking and therefore for the regulation of cell adhesion and motility (Figure 5B; 50 ng/ml: 2.91 ± 0.91; 100 ng/ml: 2.29 ± 0.53). Treatment with 1 ng/ml TGF-β2 did not lead to significant changes in α-actinin synthesis, compared to untreated control cells (Figure 5B; 1.71 ± 0.46). To evaluate the effects of TGF-β2 and CCN2/CTGF on astrocyte migration, scratch assays were carried out. Scratches were measured before and 12 h after the treatment and the migration area was calculated. After 12 h the treatment with 1 ng/ml TGF-β2 resulted in a significantly increased migration rate, in comparison to untreated cells (Figure 5C; 142.28 % ± 23.78%). Treatment with 50 ng/ml CCN2/CTGF (118.07 % ± 11.71%) or 100 ng/ml CCN2/CTGF (134.81 % ± 4.40%) also increased the migration rate significantly, compared to controls (Figure 5C).

### Extracellular Matrix Component Upregulation by Transforming Growth Factor-β2 and Cellular Communication Network Factor2/Connective Tissue Growth Factor in Murine Optic Nerve Astrocytes

**FIGURE 6:**
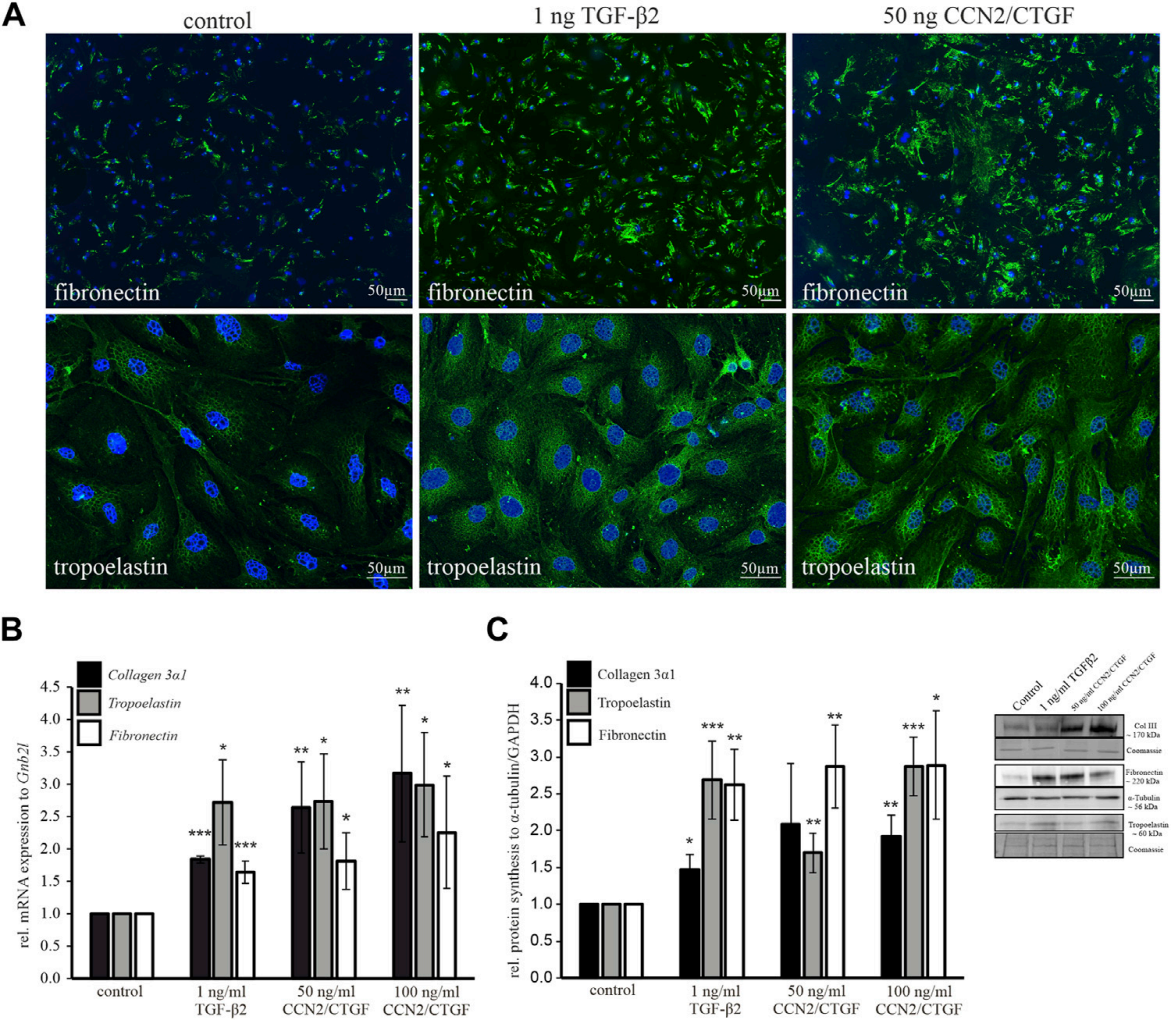
Analyses of ECM components in murine ON astrocytes following treatment with TGF-β2 and CCN2/CTGF. **(A)** Immunocytochemical staining against fibronectin (green, upper panel) showed a markedly increase following the treatment with 1 ng/ml TGF-β2 or 50 ng/ml CCN2/CTGF. Immunocytochemical staining against tropoelastin (green, lower panel) showed a pronounced increase after the treatment with 1 ng/ml TGF-β2 or 50 ng/ml CCN2/CTGF. Nuclei were stained with Dapi (blue). **(B)** Real-time RT-PCR analyses shows an intense upregulation of *collagen 3α1*, *tropoelastin* and *fibronectin* mRNA in murine ON astrocytes after treatment with 1 ng/ml TGF-β2, 50 ng/ml CCN2/CTGF or 100 ng/ml CCN2/CTGF for 24 h compared to untreated control cells (*collagen 3α1*: *n* = 3; TGF-β2 ***p = 0.0000004, 50 ngCTGF **p = 0.005, 100ngCTGF **p = 0.008; *fibronectin*: *n* = 6, TGF-β2 *p = 0.00004, 50 ng CTGF *p = 0.016, 100 ngCTGF *p = 0.05; *elastin*: *n* = 5, TGF-β2 *p = 0.026, 50 ng CTGF *0.04, 100 ng CTGF *p = 0.03). mRNA expression was normalized to *Gnb2l* and mean value of control cells was set at 1. **(C)** Western Blot analysis show an increase in protein synthesis of collagen 3α1, elastin and fibronectin in murine ON astrocytes after treatment with 1 ng/ml TGF-β2, 50 ng/ml CCN2/CTGF or 100 ng/ml CCN2/CTGF for 24 h compared to untreated control cells (collagen 3α1: *n* = 3, TGF-β2 *p = 0.03, 100 ngCTGF **p = 0.009; fibronectin: *n* = 5, **p = 0.005, 50 ngCTGF **p = 0.006, 100 ng CTGF *p = 0.02; tropoelastin: *n* = 5, TGF-β2 ***p = 0.0004, 50 ngCTGF **p = 0.002, 100 ngCTGF ***p = 0.00001). GAPDH and α-tubulin were used to normalize protein synthesis and mean value of control cells was set at 1. Right panel shows representative Western Blots for all three proteins. Data represented as mean ± SD.

Furthermore, the direct effect of CCN2/CTGF on ECM protein expression was investigated in murine ON astrocytes *in vitro*. Immunocytochemical staining, Western blot analyses and Real time RT-PCR analyses were performed for different ECM components. Immunoreactivity of fibronectin and tropoelastin showed a marked increase after treatment with TGF-β2 and CCN2/CTGF (Figure 6A). To analyze the ECM induction in a molecular approach, we investigated additionally one of the several collagen types, collagen 3a1 (Col3a1). We wanted to analyze a fibrillar collagen component. In the murine glial lamina region, the immunohistochemical staining against collagen type III and collagen type I showed an intense signal in this region (May and Lutjen-Drecoll, 2002). We focused on collagen type III as in remodeling processes collagen type III is primarily build before the stronger collagen type I (Suda et al., 2016). Murine ON astrocytes were treated again with 1 ng/ml TGF-β2, or 50 ng/ml CCN2/CTGF and 100 ng/ml CCN2/CTGF for 24 h. Treatment with TGF-β2 or CCN2/CTGF led to a significantly higher expression of mRNA for *Col3a1*, compared to untreated control cells (1 ng/ml TGF-β2: 2.30 ± 0.72, 50 ng/ml CCN2/CTGF: 2.47 ± 0.70, 100 ng/ml CCN2/CTGF: 3.07 ± 1.03; Figure 6B). In comparison to control, *fibronectin* was elevated significantly after treatments on mRNA in comparison to untreated control cells (1 ng/ml TGF-β2: 1.64 ± 0.17, 50 ng/ml CCN2/CTGF: 1.81 ± 0.44, 100 ng/ml CCN2/CTGF: 2.26 ± 0.87; Figure 6B). Finally, mRNA for *tropoelastin* was also induced significantly after the treatments with TGF-β2 or CCN2/CTGF, compared to untreated control cells (1 ng/ml TGF-β2: 2.72 ± 0.66, 50 ng/ml CCN2/CTGF: 2.74 ± 0.73, 100 ng/ml CCN2/CTGF: 2.99 ± 08; Figure 6B). The results from Western blot analyses detecting collagen type III, fibronectin and tropoelastin supported the mRNA data. Collagen type III was significantly upregulated by 1.5-fold after TGF-β2 treatment and 2-fold after CCN2/CTGF in comparison to untreated control cells (1 ng/ml TGF-β2: 1.47 ± 0.2, 50 ng/ml CCN2/CTGF: 2.08 ± 0.83, 100 ng/ml CCN2/CTGF: 1.92 ± 0.29; Figure 6C). Furthermore, fibronectin was significantly elevated after the treatments with TGF-β2 and CCN2/CTGF compared to untreated cells (1 ng/ml TGF-β2: 2.62 ± 0.48, 50 ng/ml CCN2/CTGF: 2.87 ± 0.56, 100 ng/ml CCN2/CTGF: 2.89 ± 0.74; Figure 6C). Finally, western blot experiments to detect tropoelastin, the soluble precursor of elastin showed a high and significant upregulation after the treatments in comparison to untreated control cells (1 ng/ml TGF-β2: 2.69 ± 0.53, 50 ng/ml CCN2/CTGF: 1.70 ± 0.27, 100 ng/ml CCN2/CTGF: 2.87 ± 0.4; Figure 6C).

### Increasing Substratum Stiffness Cause an Increase in Astrocyte Reactivity, Actin Cytoskeleton Modification and an Increase in Cellular Communication Network Factor 2/Connective Tissue Growth Factor

**FIGURE 7:**
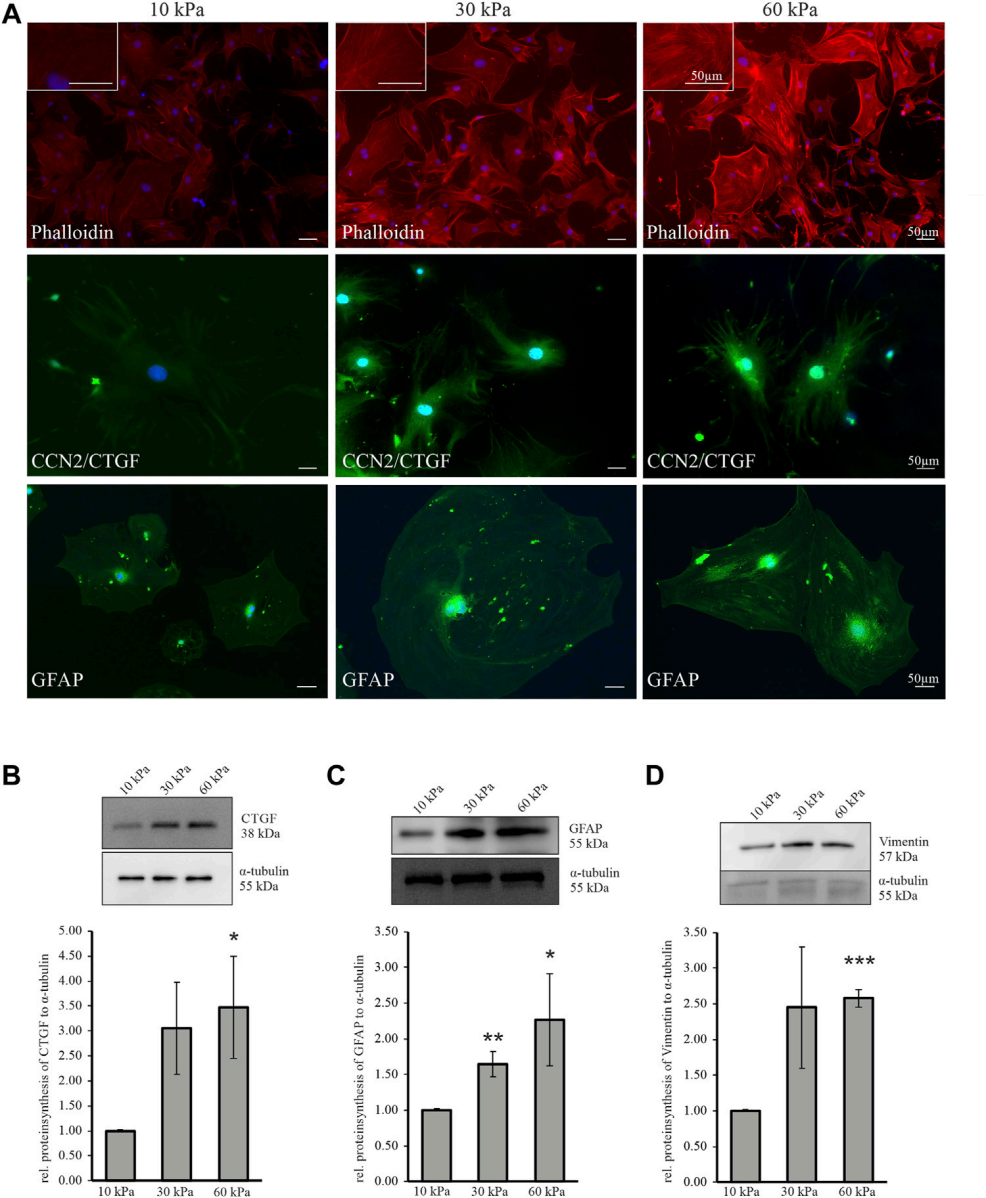
Increasing substratum stiffness causes alterations in murine ON astrocyte reactivity, actin cytoskeleton and CCN2/CTGF level. **(A)** Filamentous actin labeled by phalloidin (red, upper panel) is increased when murine ON astrocytes were cultured on substrates with higher stiffness (30 kPa, 60 kPa) compared to softer control (10 kPa). Increasing substratum stiffness leads to an enhanced formation of actin stress fibers. Immunoreactivity of CCN2/CTGF (green, middle panel) is markedly increased when murine ON astrocytes were grown on substrates with higher stiffness (30, 60 kPa) compared to softer control (10 kPa). Murine ON astrocytes show an enhanced GFAP protein synthesis (green, lower panel) when cultured on 30 or 60 kPa compared to 10 kPa. Nuclei were stained with Dapi (blue). **(B)** CCN2/CTGF protein synthesis is significantly increased in murine ON astrocytes grown on 60 kPa compared to 10 kPa (10 kPa: *n* = 5; 30 kPa: *n* = 5; 60 kPa: *n* = 5; *p = 0.04; unpaired two-tailed *t*-test). **(C)** Protein synthesis of GFAP (10 kPa: *n* = 6; 30 kPa: *n* = 6, **p = 0.005; 60 kPa: *n* = 4, *p = 0.04; unpaired two-tailed *t*-test) show an enhanced reactivity of murine ON astrocytes cultured on 30 kPa or 60 kPa compared to 10 kPa control. **(D)** Vimentin protein synthesis is significantly increased in murine ON astrocytes cultured on 60 kPa compared to 10 kPa (10 kPa: *n* = 4; 30 kPa: *n* = 4; 60 kPa: *n* = 3 ***p = 0.00002). Protein synthesis was normalized to α-tubulin and mean value of control (10 kPa) was set at 1. Data represented as mean ± SEM.

To investigate the effect of increasing substratum stiffness on mouse ON astrocytes, cells were cultured on Poly dimethylsiloxane (PDMS) substratum with different E-moduli (10 kPa, 30 kPa, 60 kPa) (see Supplementary Methods S2; Supplementary Figure S2). First, the actin stress fibers were labeled with phalloidin to assess if astrocytes can sense the changes in stiffness of their surrounding matrix and visualize the effect on the actin cytoskeleton structure. We could observe an intensification of the labeled F-actin filaments with increasing stiffness (Figure 7A, upper panel). Additionally, the increased substratum stiffness caused an increase in longitudinally-oriented actin stress fibers. Astrocytes grown on a substratum with the stiffness of 60 kPa contained numerous longitudinally arranged actin stress fibers (Figure 7A, upper panel), which were longer and thicker compared to those grown on substratum with 10 kPa or 30 kPa (Figure 7A, upper panel). Next, we performed immunocytochemial staining against CCN2/CTGF and GFAP. CCN2/CTGF immunoreactivity was increased in astrocytes grown on stiffer substratum, compared to cells cultured on 10 kPa (Figure 7A, middle panel). Similar results were seen for GFAP, which was more intense in astrocytes grown on 30 or 60 kPa compared to those cultured on 10 kPa (Figure 7A, lower panel). To further evaluate the effect of increasing substratum stiffness on murine ON astrocytes we performed western blot analyses against CCN2/CTGF, GFAP and Vimentin. Western blotting experiments of proteins from astrocytes grown on a substratum with an E-modulus of 60 kPa showed an increase in the amounts of CCN2/CTGF (60 kPa: 3.48 ± 1.02), compared with proteins from astrocytes cultured on substrata with an E-modulus with 10 kPa (Figure 7B). In cells cultured on 30 kPa enhanced, but not significant increase for CTGF was detected (30 kPa: 3.05 ± 0.92).In proteins from cells grown on substratum with a 30 kPa or 60 kPa stiffness, we observed significantly increased amounts of GFAP compared to those from cells grown on 10 kPa (30 kPa: 1.65 ± 0.18; 60 kPa: 2.27 ± 0.64; Figure 7C). Vimentin protein was also significantly increased when cells were grown on the 60 kPa substratum compared to astrocytes plated on 10 kPa (60 kPa: 2.58 ± 0.12; Figure 7D). In cells cultured on 30 kPa enhanced, but not significant, increase for Vimentin was detected (30 kPa: 2.45 ± 0.85).

## Discussion

We conclude that the transgenic βb1-CTGF1 mouse can be used as a model for primary open-angle glaucoma (POAG) to study pathologic changes in the ONH. This conclusion rests on the enhanced astrocytic reactivity in the ONH and the increased ECM protein synthesis in the peripapillary sclera during the pathologic development including increased IOP and an ascending axonal loss in the ONH. Further we conclude that *Ccn2/Ctgf* expression in the ONH is driven by the biomechanical alterations occurring during POAG, causing an augmentation of pathogenic effects in the glaucomatous ONH. The conclusion is based on I) the increased *Ccn2/Ctgf* expression in the glial lamina of βB1-CTGF1 mice during the progression of the axon loss, II) the reinforced appearance of CCN2/CTGF in the lamina cribrosa of glaucomatous patients, III) the finding that CCN2/CTGF treatment provokes an increased synthesis of GFAP and ECM proteins in astrocytes *in vitro* and IV) the observation that CCN2/CTGF levels can be modulated by biomechanical alteration like stiffness.

The understanding of the pathological mechanisms in glaucoma was greatly enhanced by the establishment of different mouse models. The opportunity to study alterations in the ONH tissues under increased IOP conditions *in vivo* are necessary to get a fundamental knowledge about molecular, cellular and biomechanical mechanisms contributing to the axonal damage in glaucoma. In this study we characterized the ONH region of the βB1-CTGF1 mouse, a potential transgenic mouse model for glaucoma. The transgenic overexpression of CCN2/CTGF in the mouse eye causes an increase in IOP and a continuous decline in the number of ON axons and RGCs, which is accompanied by an increased reactivity of retinal astrocytes and Müller cells (Junglas et al., 2012; Reinehr et al., 2019). In this study we could observe an increased GFAP immunoreactivity in astrocytes of the glial lamina, an enhancement of phalloidin-labeled filamentous actin in the ONH and higher amounts of fibronectin in the peripapillary sclera *in situ* in βB1-CTGF1 mice.

In POAG similar mechanisms are taking place like ECM changes in the lamina cribrosa and the peripapillary sclera coming along with increased GFAP synthesis in the ONH (Hernandez and Pena, 1997; Kerr et al., 2011). There is considerable evidence that an increase in IOP leads to changes in the biomechanical properties of the sclera, an assumption that is supported by the observation that chronically elevated IOP results in an increased scleral stiffness in mouse and monkey, quite similar to that what is found in human glaucomatous eyes (Downs et al., 2005; Nguyen et al., 2013). Mechanical tension in POAG appears to be increased in both the lamina cribrosa and the peripapillary sclera. A comparison of the stress/strain relationship in the sclera of different species demonstrated that the greatest strain is in the peripapillary sclera (Coudrillier et al., 2012). The biomechanical nature of the peripapillary sclera is especially important in the mouse ONH, where a lamina cribrosa with connective tissue lamellae is missing. Instead, astrocytes of this region form a glial lamina that consists of astrocytes whose processes are in direct contact with the peripapillary sclera (Sun et al., 2009). ONH astrocytes are capable to directly transfer scleral wall tension to passing ON axons (Quigley and Cone, 2013). In the 2 month-old βB1-CTGF1 mouse we could observe changes in cellular morphology of the ONH astrocytes and changes in the expression pattern like in other chronic and acute glaucoma mouse models after an increase of IOP. The initial biomechanical insult to the ONH after IOP elevation caused astrocyte reactivity accompanied by a significant reorganization of the phalloidin-labeled filamentous actin in the ONH (Tehrani et al., 2019). A similar effect was observed in the 2 month-old βb1-CTGF1 mouse, where in the glial lamina an enhanced formation of filamentous actin was detected. Former analysis of human ONH astrocytes under biomechanical strain revealed profound changes in the synthesis pattern including the TGF-β pathway (Rogers et al., 2012). Furthermore, the expression of mechanosensitive channels in astrocytes was already proven (Choi et al., 2015). *In situ* results of this study show that there is a direct connection between biomechanical load and the expression of CCN2/CTGF in astrocytes of the ONH region. The astrocytes of the prelaminar region of the glial lamina site showed a strong signal for the CCN2/CTGF promoter activity, whereas the astrocytes of the postlaminar region and the retinal astrocytes exhibit only a faint staining (Dillinger et al., 2021). Our findings clearly support that astrocytes can sense mechanical cues in their ambient substratum and react on biomechanical alterations of the surrounding environment with changes in GFAP, vimentin and CCN2/CTGF synthesis. Additionally, the increased CCN2/CTGF levels in the periphery of the glaucomatous human ON, but not the central part supports the finding that the lamina cribrosa strains were larger in the peripheral lamina cribrosa compared to the central lamina cribrosa, and lamina cribrosa strains in the more severely damage glaucoma group were larger than those in the more mildly damaged group and larger differences were measured between peripheral and central lamina cribrosa strains in the more severely damaged glaucoma group (Midgett et al., 2020). The increased lamina cribrosa strain can be sensed by ON cells, like astrocytes and/or lamina cribrosa cells. It is of interest to note that the ON astrocytes can sense small changes in the range of kPa as the glaucomatous changes occurring in the lamina cribrosa are suggested to be of a much higher magnitude (MPa) (Spoerl et al., 2005), leading to the assumption that at the onset of the disease small alterations of biomechanical properties could be sensed by the astrocytes inducing changes in their expression patterns. A recent finding described that the inhibition of mechanosensitive channels attenuated the TGF-β2 mediated GFAP expression and actin cytoskeleton remodeling in ON astrocytes, pointing also into the direction that the mechanosensitive channels are involved at the early phase of the disease (Kirschner et al., 2021).

The ECM changes in the ONH region appear to be due to a disruption of the homeostatic balance of growth factors in the ONH region (Fuchshofer, 2011). One of the identified growth factors is TGF-β2, which was shown to be elevated in the ONH and aqueous humor of patients suffering from POAG (Tripathi et al., 1994; Pena et al., 1999; Picht et al., 2001; Zode et al., 2011). In the course of various diseases, increased levels of TGF-β1 and 2 contribute to fibrotic processes in many tissues (Pohlers et al., 2009) and analog mechanisms are responsible for the ECM changes that occur in the lamina cribrosa of POAG patients (Pena et al., 2001). The results of our study strongly support the concept that reactive astrocytes are involved in the disruption of the homeostatic balance of growth factors, as CCN2/CTGF was found be upregulated in the glaucomatous human ONH and in the ONH of the βb1-CTGF1 mouse. CCN2/CTGF, a matricellular protein of the CCN family, is the downstream mediator of the TGF-β2 mediated fibrotic effect. Accordingly, silencing of CCN2/CTGF in human ONH astrocytes prevented the increase of ECM proteins following TGF-β2 treatment, leading to the assumption that CCN2/CTGF is an essential factor contributing to the glaucomatous changes in the ONH of POAG patients (Fuchshofer et al., 2005). Hitherto, the effect of increased CCN2/CTGF levels on ONH astrocytes has not been investigated. The increased migration rate of murine ON astrocytes and the increased ECM and GFAP synthesis after treatment with CCN2/CTGF points towards the capacity of CCN2/CTGF to induce a reactive phenotype in astrocytes. It is of interest that the murine ON astrocytes reacted in a similar manner as the human ONH astrocytes regarding the stimulation with CCN2/CTGF or TGF-β 2. We cannot exclude that the differences in the astrocytic reaction between ON and ONH astrocytes could be seen after different treatments.

In glaucomatous eyes, lamina cribrosa astrocytes were observed to migrate into nerve bundles of the ON (Hernandez and Pena, 1997; Varela and Hernandez, 1997; Hernandez, 2000). Reactive astrocytes migrate towards the insult, which is thought as an attempt to limit secondary damages by increased synthesis of ECM proteins and the formation of a glial scar. In POAG, the gradual loss of axons is known to involve a gliosis reaction and the remodeling of the lamina cribrosa at the ONH (Hernandez, 2000; Morrison, 2006). Changes in ECM production are very likely linked to an increased IOP in various animal models since they were observed in primate and rodent glaucoma models (Morrison et al., 1990; Johnson et al., 1996; Johnson et al., 2000; May and Mittag, 2006). Quite intriguingly, a microarray analysis of the ONH after inducing experimental glaucoma in rats demonstrated that not only ECM proteins are upregulated, but also members of the TGF-β superfamily, such as TGF-β1 (Johnson et al., 2007). Since CCN2/CTGF is a downstream mediator of TGF-βs, it is likely that the observed upregulation of TGF-β1 causes an increase in CCN2/CTGF synthesis.

In the present study we found an increase of CCN2/CTGF in the ONH of the transgenic mouse model at 2 months of age. At that age, the mice suffer from an increased IOP and a significant loss of axons in the ON in comparison to wild-type littermates (Junglas et al., 2012). Based on our *in situ* and *in vitro* findings, it appears to be likely that astrocytes are the source of the increasing amounts of CCN2/CTGF. Since the transgenic mouse model that was used in this study, is based on a lens-specific expression of CCN2/CTGF in the anterior eye, we could exclude the possibility that the observed increase of CCN2/CTGF is due to the transgenic overexpression of CCN2/CTGF from the lens as the highest activity of the βb1-crystallin promoter is during the development of the lens and the analysis of the glial lamina of 1 month-old transgenic animals did not show any increase of CCN2/CTGF immunoreactivity in the ONH region. Therefore, we could also exclude the autocrine induction of CCN2/CTGF (Junglas et al., 2009), as the induction would be at the highest level, when the promoter has the highest activity. Instead, we observed that the mRNA expression of *Ccn2/Ctgf* was not altered between the 1 month-old transgenic and wildtype littermates, whereas in the 2 month-old animals an increased *Ccn2/Ctgf* mRNA expression was observed in the transgenic animals. The increase of CCN2/CTGF in the glial lamina of the transgenic animals was associated with a reactive phenotype in the resident astrocytes, indicated by the increase of their GFAP immunoreactivity and a thickening of their processes. Similar findings were described in the ONH of decorin knockout mice, where morphological changes of astrocytes in the glial lamina caused by an increased IOP were accompanied by increased GFAP and CTGF/CCN2 synthesis in the ONH (Schneider et al., 2021a; Schneider et al., 2021b). An upregulation of CCN2/CTGF was observed in reactive astrocytes during other neurodegenerative diseases like multiple sclerosis (Holley et al., 2003), amyotrophic lateral sclerosis (Spliet et al., 2003) and Alzheimer’s disease (Ueberham et al., 2003). Furthermore, an upregulation of CCN2/CTGF was found to be associated with reactive gliosis in stab-wounded rat brains or in the brain of stroke patients (Schwab et al., 2000; Schwab et al., 2001). In the βB1-CTGF1 mouse, minor amounts of fibronectin were found deposited within the glial lamina of the ON, a finding that was not observed in wild-type littermates. In contrast, the βB1-CTGF1 mouse showed a significant increase of fibronectin in the peripapillary sclera. The biomechanical properties of the peripapillary sclera are dependent on its specific molecular components and increasing amounts of fibronectin may contribute to an increased stiffness. The parallel observation of increased amounts of filamentous actin within the glial lamina of the βB1-CTGF1 mice is also expected to alter the biomechanical properties in the glial lamina region.

It is tempting to speculate that reactive changes in ONH astrocytes induced by the altered biomechanical characteristics of the region give rise to a self-amplifying process that includes increased TGF-β2/CCN2/CTGF signaling, leading to the synthesis of ECM molecules and cytoskeletal proteins, a process that in turn augments the stiffness at the ONH. Such a scenario may finally result in a vicious circle as the causative mechanism for ONH deformation in POAG

## Materials and Methods

### Animals

Transgenic βB1-CTGF1 mice were generated as described previously (Junglas et al., 2012) and compared to wildtype mice. Mice were housed under standardized conditions of 62% air humidity and 21°C room temperature. Feeding was *ad libitum*. Animals were kept at a 12 h light/dark cycle (6 am–6 pm). All procedures conformed to the tenets of the National Institutes of Health Guidelines on the Care and Use of Animals in Research, the EU Directive 2010/63/E and the ARVO Statement for the Use of Animals in Ophthalmic and Vision Research, and were approved by the local authorities (54-2532.1-44/12; Regierung Oberpfalz, Bavaria, Germany).

### RNA Analysis

Astrocytes were cultured either on 6-well plates and treated with 1 ng/ml TGF-β2, 50 ng/ml CCN2/CTGF or 100 ng/ml CCN2/CTGF, or cultured on PMDS substrata with different E-moduli (10kPa, 30 kPa, 60 kPa). The concentration for CCN2/CTGF treatment were chosen in accordance to our previous findings (Junglas et al., 2009).Before enucleation of the eyes, mice were anesthetized with CO_2_ and euthanized by atlanto-occipital dislocation. First the anterior eye segment, the lens and retina were removed, and the ON was cleaned from muscles and connective tissue. The sclera was cut away from the ONH and potentially remaining parts of sclera and retina were removed. Second the ON tissue was separated from the ONH. Both tissue ON and ONH were processes independently for following experiments. Total RNA was extracted with TriFast™ (Peqlab, Erlangen, Germany) according to the manufacture’s recommendations. cDNA was prepared from total RNA using the qScript™cDNA Synthesis Kit (Quanta Biosciences, Gaithersburg, United States) according to the manufacturer’s introductions. Real-time RT-PCR was performed on a BioRad iQ5 Real-Time PCR Detection System (BioRad, München, Germany) with the temperature profile as follows: 50 cycles of 20 s melting at 94°C, 10 s of annealing at 60°C and 20 s of extension at 60°C. All primers were purchased from Invitrogen and extended over exon-intron boundaries: 5′-ccc​ctg​gaa​tct​gtg​aat​c-3′ (*msCol3a1* forward), 5′-tga​gtc​gaa​ttg​ggg​aga​at-3′ (*msCol3a1* reverse), 5′-gct​gct​gct​aag​gct​gct​aa-3′ (*msElastin* forward), 5′-agc​acc​tgg​gag​cct​aac​tc-3′ (*msElastin* reverse), 5′-cgg​aga​gag​tgc​ccc​tac​ta-3′ (*msFN* forward), 5′-cga​tat​tgg​tga​atc​gca​ga-3′ (*msFn* reverse), 5′-tct​gca​agt​aca​cgg​tcc​ag-3′ (*msGnb2l* forward), 5′-gag​acg​atg​ata​ggg​ttg​ctg-3′ (*msGnb2l* reverse), 5′-tga​cct​gga​gga​aaa​cat​taa​ga-3′ (*msCcn2/Ctgf* forward), 5′-agc​cct​gta​tgt​ctt​cac​act​g-3′ (*msCcn2/Ctgf* reverse), 5′-tcg​aga​tcg​cca​cct​aca​g-3′ (*msGfap* forward), 5′-gtc​tgt​aca​gga​atg​gtg​atg​c-3′ (*msGfap* reverse), 5′-gct​gcc​atc​tgt​ttt​acg​g-3′ (*msRpl32* forward), 5′-tga​ctg​gtg​cct​gat​gaa​ct-3 (*msRpl32* reverse) or Taqman^®^ probes were used (Rpl32: Mm02528467_g1; Gfap: Mm01253033_m1; Ccn2/Ctgf: Mm01192933_g1 from ThermoFisher Scientific, Darmstadt, Germany). RNA that was not reverse transcribed served as negative control. For relative quantification of the experiments, *Gnb2l* and *Rpl32* were used as a housekeeping gene. BioRad iQ5 Optical System Software (version 2.0) was used for analysis and ΔΔct-method was applied for normalization.

### Western Blot Analysis

Proteins were isolated following the RNA isolation according to the manufacturer’s instructions. The proteins were dissolved in 1% SDS containing protease (Serva Electrophoresis GmbH, Heidelberg, Germany) and phosphatase inhibitor (Sigma-Aldrich, Taufkirchen, Germany). Protein concentration was determined by the bicinchoninic acid assay (Interchim, Montluçon Cedex, France). Proteins were separated by SDS-PAGE and transferred to polyvinylidene difluoride (PVDF) membranes (Roche, Mannheim, Germany). Western Blot analysis was performed with specific antibodies as described previously (Fuchshofer et al., 2005). Specific antibodies were used as follows: goat anti-α-actinin (1:500), rabbit anti-Col III (1:250; Santa Cruz Biotechnology, Cat# sc-28888, RRID:AB_2082354), goat anti-CTGF (1:500; Santa Cruz Biotechnology, Cat# sc-14939, RRID:AB_638805), rabbit anti-fibronectin (1:500; Santa Cruz Biotechnology, Cat# sc-9068, RRID:AB_2105699), chicken anti-GFAP (1:10,000; LSBio (LifeSpan), Cat# LS-B4775-50, RRID:AB_10803257), rabbit anti-tropoelastin (1:250, Elastin Products Company, Owensville, MO, United States), goat anti-vimentin (1:500, Sigma-Aldrich Cat# V4630, RRID:AB_477619, Taufkirchen, Germany), donkey anti-goat (1:2000, Bethyl Laboratories Inc., Montgomery, TX, United States, Cat# A50-201P, RRID:AB_66756), goat anti-rabbit (1:5,000, Cell Signaling Technology, Danvers, MA, United States, Cat# 7074, RRID:AB_2099233), rabbit anti-chicken (1:2,000, Bethyl Laboratories Inc., Montgomery, TX, United States, Cat# A30-207P, RRID:AB_67387). Chemiluminescence was detected on a LAS 3000 imaging workstation (Fujifilm, Düsseldorf, Germany). GAPDH (rabbit anti-GAPDH-HRP, 1:5,000, Cell Signaling Technology, Cat# 3683, RRID:AB_1642205) or α-tubulin (rabbit anti-α-tubulin, 1:2500, Rockland Immunochemicals Inc., Gilbertsville, United States, Cat# 600-401-880, RRID:AB_2137000) were used as loading controls to normalize the signal intensity of the Western blots. The intensity of the bands detected by Western blot analysis was determined using appropriate software (AIDA Image analyzer software, Raytest).

### Immunocytochemistry

Astrocytes were plated on 35 mm µ-dishes (ibidi, Martinsried, Germany) with different E-moduli (10 kPa, 30 kPa, 60 kPa) at a density of 20000 cells per dish for phalloidin labeling, GFAP and CCN2/CTGF staining (Cells were cultured 7 days and starved under serum free conditions 24 h before fixation). For the characterization of the isolated ON astrocytes and for analysis of the effect of CCN2/CTGF treatment on the ECM synthesis, cells were seeded on coverslips and treated with either 1 ng/ml TGF-β2, 50 ng/ml CCN2/CTGF or left untreated as controls for 24 h under serum free conditions. Cells were washed twice with PBS, fixed with 4% (w/v) paraformaldehyde (PFA) for 5 min and washed again three times with PBS. Specific antibodies for staining were used as follows: rabbit anti-CTGF (1:200, Genetex, Irvine, United States, Cat# GTX26992, RRID:AB_369067), rabbit anti-GFAP (1:100, Dako, Hamburg, Germany, Agilent Cat# Z0334, RRID:AB_1001338), rabbit anti-tropoelastin (1:20, Elastin Products Company, Owensville, MO, United States), rabbit anti-fibronectin (1:100, Santa Cruz, Dallas, TX, United States, Cat# sc-9068, RRID:AB_2105699), Alexa Fluor 488-conjugated anti rabbit IgG (1:1,000, Invitrogen, Darmstadt, Germany, Thermo Fisher Scientific Cat# A-11070, RRID:AB_2534114) and Cy3™ goat anti rabbit (1:2000, Jackson Immuno Research Europe Ltd., Suffolk, United Kingdom, Cat# 111-165-144, RRID:AB_2338006). Phalloidin-rhodamin (1:1,000, 1 h at room temperature, Sigma-Aldrich, Taufkirchen, Germany) was used for labeling the actin cytoskeleton. As a control for unspecific binding of secondary antibodies, negative controls were performed. Finally, 4,6-diamidino-2-phenylindole (DAPI) (Vector Laboratories, Burlingame, CA, United States) diluted 1:10 in PBS was added to counterstain nuclear DNA and the immunofluorescence was visualized using a Zeiss Axio Imager fluorescence microscope (Carl Zeiss AG, Göttingen, Germany).

### Migration Assay

Astrocytes were grown to confluence in 6-well plates. 24 h prior to treatments, cells were starved under serum-free conditions. Three equal scratches were made in the cell layer of each well. To suppress proliferation, cells were treated with 5 μg/ml Mitomycin C (Sigma-Aldrich). Cells were treated with 1 ng/ml TGF-β2, 50 ng/ml CTGF or 100 ng/ml CTGF, untreated cells were used as control. Pictures were taken directly before (0 h) and 12 h after the treatment. For each time point, length and region of the scratches were measured using Axiovision 4.8 (Carl Zeiss AG). Region of migration (ΔA) was counted by subtracting the region at 12 h (At) from the region at 0 h (A0). To correct the results, they were then divided by the length of the scratch 0 h (A0-At)/s = ΔA/s. Migration assays were carried out several times. Results were allocated to an arithmetic mean.

### Immunohistochemistry

Methods for securing human tissue were humane, included proper consent and approval, and complied with the Declaration of Helsinki. Human ON were obtained from two healthy and four glaucomatous eyes. Human ON were embedded in paraffin and 6 µm sections were deparaffinized and washed in water. For immunohistochemistry ON sections were pretreated with proteinase K followed by blocking with 2% bovine serum albumin (BSA), 0.2% cold water fish gelatin (Sigma-Aldrich). Sections were incubated with rabbit anti-CTGF (1:50, novusbio, Centennial, CO, United States, Cat# NB100-724SS, RRID:AB_921075) at 4°C overnight. Afterwards sections were washed three times with 0.1 M phosphate buffer, followed by the incubation with Alexa Flour ^®^ 647 donkey anti-rabbit (1:200, Thermo Fisher Scientific Cat# A-31573, RRID:AB_2536183) for 2 h at room temperature. As a control for unspecific binding of secondary antibodies, negative controls performed. After washing three times with 0.1 M phosphate buffer, the slides were mounted using the DakoCytomation fluorescent mounting medium with DAPI 1:10 (Dako). Slides were dried overnight at 4°C before microscopy. Eyes of βB1-CTGF1 transgenic (TG) and wildtype (WT) mice were enucleated and fixed in 4% (w/v) paraformaldehyde (PFA) for 24 h. The eyes were equilibrated in 10%, 20% and 30% sucrose, embedded in Tissue Tek optimal cooling temperature compound (Sakura Finetek Europe B.V., Zoeterwounde, Netherland), and stored at −20°C. Frozen 12 µm sections were cut on the cryostat. Tangential sections of the glial lamina were obtained. After blocking with 1% BSA, 0.2% cold water fish skin gelatin (Sigma-Aldrich), 0.1% Triton-X-100 in 0.1 M phosphate buffer, frozen sections were incubated with rabbit anti-GFAP (1:1,000, Dako Denmark A/S,Glostrup, Denmark, Agilent Cat# Z0334, RRID:AB_1001338), rabbit anti-CTGF (1:400, Genetex Inc.,, Irvine, CA, United States, Cat# GTX26992, RRID:AB_369067), rabbit anti-fibronectin (1:500, Agilent Cat# A024502, RRID:AB_578510), chicken anti-GFAP (1:2000, LSBio (LifeSpan), Cat# LS-B4775-50, RRID:AB_10803257) or with phalloidin-rhodamin (1:500, 1 h at room temperature, Sigma-Aldrich) to label the actin cytoskeleton at 4°C overnight. Afterwards, tissue sections were washed three times with 0.1 M phosphate buffer, followed by an incubation with the secondary antibody, Alexa Flour ^®^ 488 goat anti rabbit (1:1,000, Life Technologies, Carlsbad, CA, United States, Thermo Fisher Scientific Cat# A-11070, RRID:AB_2534114), Alexa Flour ^®^ 488 goat anti chicken (1:1,000, Thermo Fisher Scientific Cat# A-11039, RRID:AB_2534096) or Cy3™ goat anti rabbit (1:2,000, Jackson Immuno Research Europe Ltd., Suffolk, United Kingdom, Cat# 111-165-144, RRID:AB_2338006) for 1 h at room temperature. As a control for unspecific binding of secondary antibodies, negative controls were performed. After washing three times with 0.1 M phosphate buffer, the slides were mounted using the DakoCytomation fluorescent mounting medium with DAPI 1:10 (Dako). Slides were dried overnight at 4°C before microscopy.

### Image Analysis

The images were analyzed using ImageJ’s built-in measuring feature (Wayne Rasband, formerly National Institutes of Health, Bethesda, MD, United States). The surface area of the ONH section was calculated and the amount of area emitting fluorescent signal for GFAP, CCN2/CTGF or filamentous actin within the outlines of the ONH section was determined by a standardized macro routine consisting of ImageJ’s color threshold plugin and particle analyzer. The resulting values were used to calculate the percentage of area within the ONH section. For fibronectin quantification, the surface area of the peripapillary sclera was quantified and the same methods of measurement that were mentioned above were used. These measurements were performed for each individual specimen after calibrating ImageJ with the scale bar. The resulting data was analyzed using SPSS (IBM, Armonk, NY, United States).

### Statistical Analysis

Western Blot and real-time RT-PCR was repeated at least three times with RNA and protein extract from mouse ON astrocytes and ON/ONH tissue. Each real-time RT-PCR analysis was performed in triplicates. The data is represented as mean ± SEM or otherwise stated in the figure legends. Statistical analysis of data was performed by two-tailed *t*-test compared to theoretical mean of 1 (normalized control, indicated in the figure legends).

## Data Availability

The raw data supporting the conclusion of this article will be made available by the authors, without undue reservation.
