# Supplementary material for: CCN2/CTGF—A Modulator of the Optic Nerve Head Astrocyte
Source: Front Cell Dev Biol. 2022 Apr 14;10:864433. doi: 10.3389/fcell.2022.864433 (PMC9047870; doi:10.3389/fcell.2022.864433)
Supplement: Supplementary file 1 [file Table1.DOCX]

| **Mixtures** | **Component** | **Parts** |
| --- | --- | --- |
| Basic elastomer mixture (BE1) | VS100 000 | 14 g |
|  | MV2000 | 2.5 g |
|  | AK10 | 44 g |
|  | Inhibitor DVS | 250 µl |
|  | Modifier 700 | 50 µl |
| 10 kPa | BE1 | 12 g |
|  | AB116655 | 0.38 g |
|  | Catalyst 510 | 40 µl |
| 30 kPa | BE1 | 12 g |
|  | Cross linker  AB116655 | 0.38 g |
|  | Cross linker 210 | 5 µl |
|  | Catalyst 510 | 40 µl |
|  | Inhibitor DVS | 10 µl |
| 60 kPa | BE1 | 12 g |
|  | Cross linker  AB116655 | 0.38 g |
|  | Cross linker 210 | 20 µl |
|  | Catalyst 510 | 70 µl |
|  | Inhibitor DVS | 70 µl |

Table 1: Elastomer mixtures of the cell substrata, showing the parameters of the basic elastomer mixture and the additional values for the different substrata
