# Supplementary figures and images for: CCN2/CTGF—A Modulator of the Optic Nerve Head Astrocyte
Source: Front Cell Dev Biol. 2022 Apr 14;10:864433. doi: 10.3389/fcell.2022.864433 (PMC9047870; doi:10.3389/fcell.2022.864433)

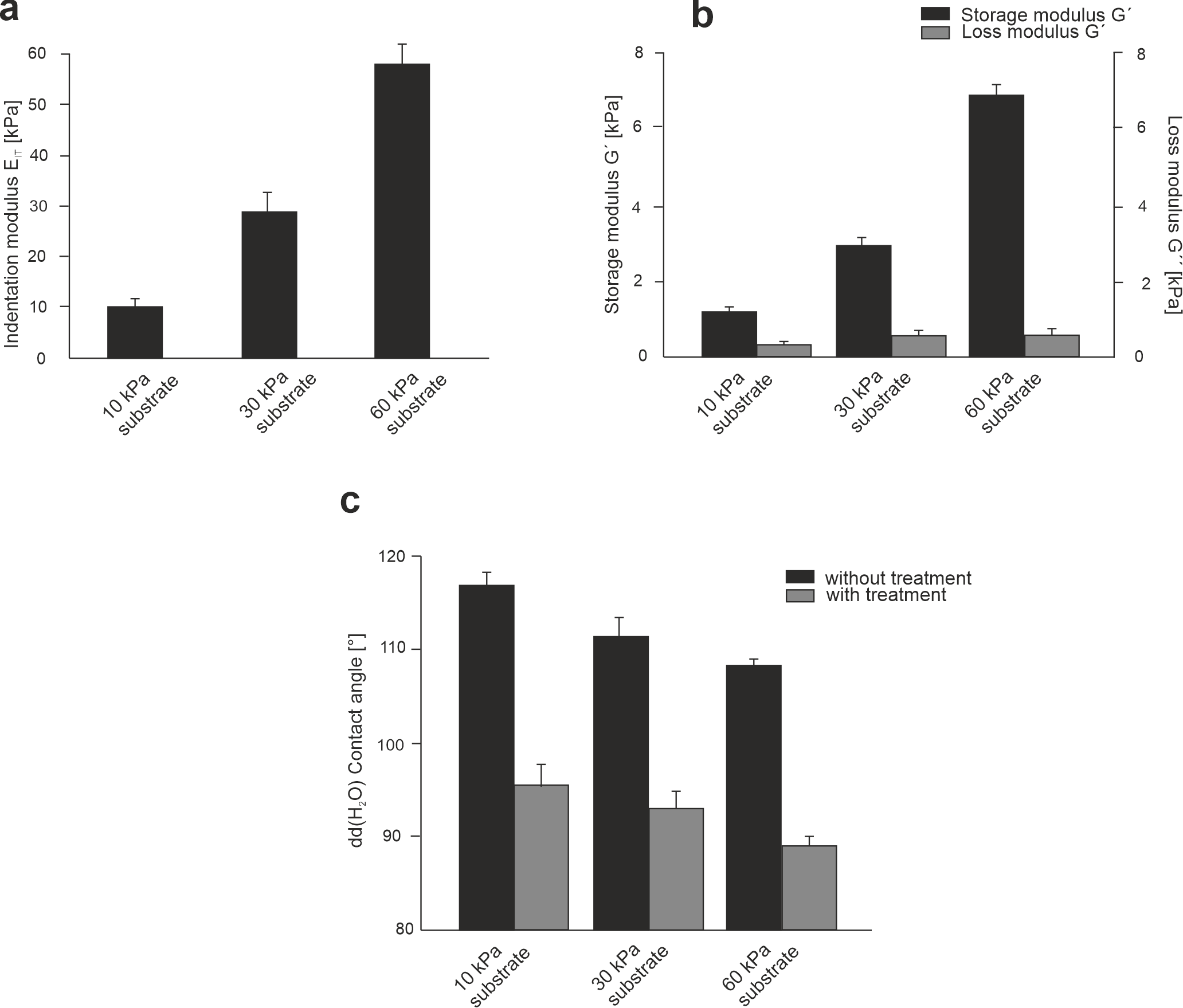

Supplement: Supplementary file 2 [file Image2.TIF]

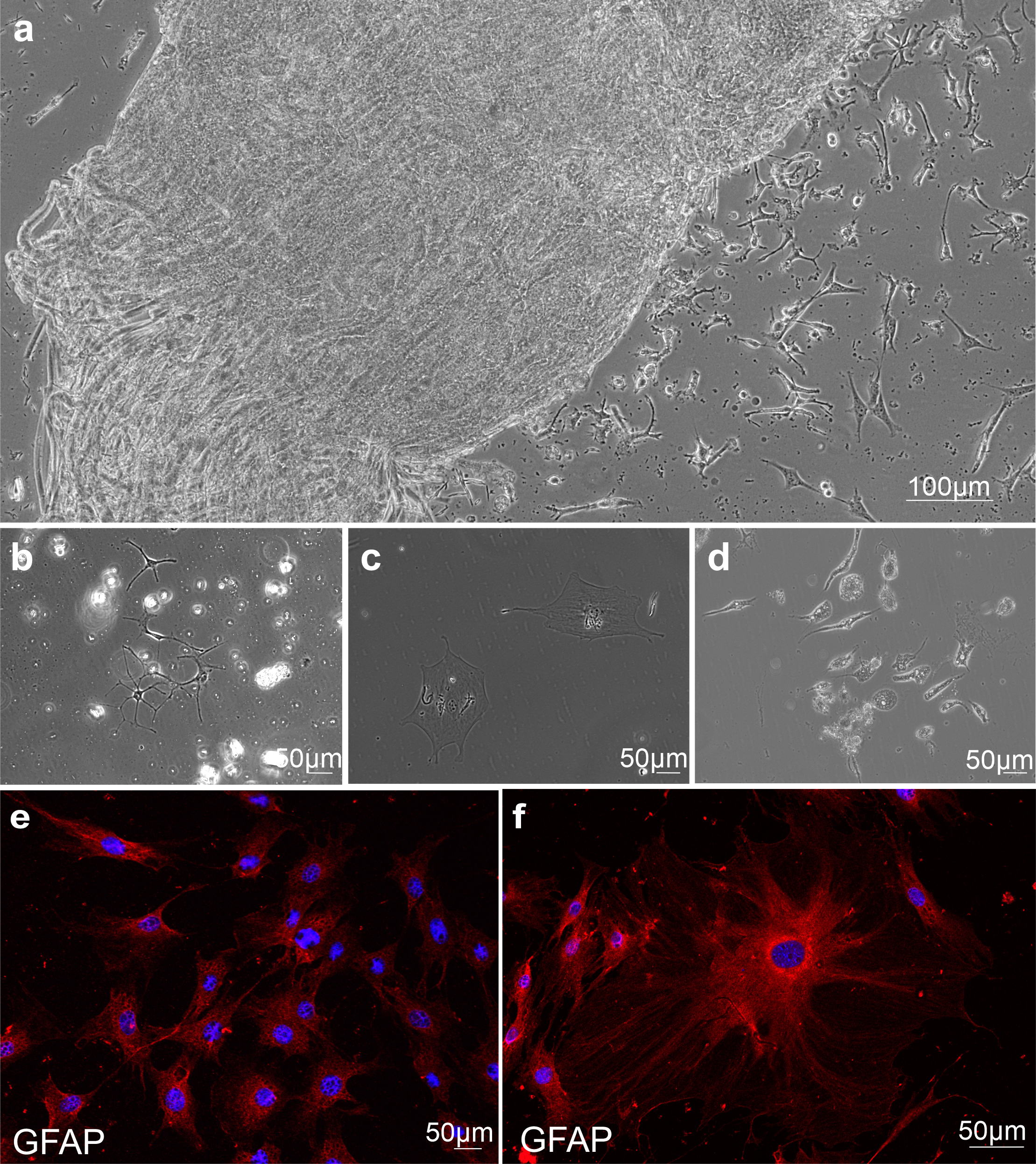

Supplement: Supplementary file 3 [file Image1.TIF]
