# Supplementary material for: CCN2/CTGF—A Modulator of the Optic Nerve Head Astrocyte
Source: Front Cell Dev Biol. 2022 Apr 14;10:864433. doi: 10.3389/fcell.2022.864433 (PMC9047870; doi:10.3389/fcell.2022.864433)
Supplement: Supplementary file 4 [file DataSheet1.docx]

Supplementary Material

# Supplementary Methods

**Isolation of primary murine optic nerve astrocytes**

CD1 mice were used for the isolation of ON astrocytes. After mice were sacrificed, both eyes were enucleated, and the ONs were cut off the globe. One murine ON astrocytes cell culture sample includes the entire ON of both eyes. After removal of the dura, ON samples were digested in 200 µl Trypsin (Gibco BRL, Karlsruhe, Germany) for 30 min at 37°C. The tissue was then sheared by repeated pipetting and plated on laminin coated 6-well plates. Cells were grown in DMEM/F12 (Gibco BRL) enriched with 10% fetal bovine serum (FBS, Gibco BRL), 1% penicillin/streptomycin (Gibco BRL) and 1% astrocyte growth supplement (Sciencell, Carlsbad, CA, UA). Medium was not changed in the following seven days to allow the cells to attach to the tissue culture plates. After seven days, the medium was replaced two times a week with fresh medium. A pure astrocyte culture was obtained by shaking the wells for 12h to remove less adhesive cells. Cells were maintained in an incubator at 37°C and 5%CO_2_. After cells grew to confluence, they were seeded in 25 cm^2^ cell culture flasks (Nunc, VWR, Darmstadt, Germany). Astrocytes were characterized via GFAP staining. Only cells from passage 2 to 10 were used for experiments.

**Polydimethylsiloxane Cell Substrata Preparation**

The addition-curing basic polymer VS100000 (vinyl-functional polydimethylsiloxane), the chain extender modifier 700 (SIH-terminated polydimethylsiloxane, difunctional structure), the reactive diluent MV2000 (mono-vinyl functional polydimethylsiloxane) and the inhibitor DVS were purchased from Evonik Hanse GmbH, Gesthach, Germany. In the first step, all the components together with the plasticizer silicone oil (linear, non-reactive polydimethylsiloxane) AK10 (Wacker Chemie AG, Munich, Germany) were rotationally mixed for 6min at 1000RPM (Roti® Speed mixing tool, Carl Roth GmbH, Germany) to form a basic elastomer (BE1) mixture. The mixing ratio of BE1 is shown in Table 2. In the next step, three different elastomers were prepared. All substrata (nominal indentation modulus *E*_IT_ = 10 kPa, 30 kPa, 60 kPa) based on the BE1 (12g) were mixed with a cross linker AB116655 0.5-1% methylhydrosiloxane–dimethylsiloxane copolymer (ABCR GmbH, Karlsruhe, Germany), and a Pt-catalyst 510 (Evonik Hanse GmbH) in a defined mixture ratio. The “30kPa” and “60kPa” samples differ only in a small amount of additional cross linker 210 (Evonik Hanse GmbH) and a necessary additional catalyst and inhibitor ratio (Table 2). Depending on the batch, the parts corresponding to standard values must be slightly modified in order to precisely achieve target mechanical properties. In addition, air bubbles were removed by placing the elastomers in a vacuum chamber for 15 min. In conclusion, Petri dishes (µ-dish 35 mm high, uncoated, ibidi, Martinsried, Germany) and Petri dishes (greiner bio-one 94x16 mm PS) were coated (ibidi 0.5 g/greiner 4.5 g) with the finished, but uncured elastomer. In a further step, remaining air bubbles in the coating were removed using a vacuum chamber for 3 min. The substrata were then cured in a universal oven (Memmert UF30Plus, Memmert GmbH, Schwabach, Germany) at 80°C for 1h and finally cured at 65°C 24h (with forced air circulation). The finished coated petri dishes were then washed twice with 2-propanol 70% (Carl Roth GmbH, Germany) for 5min and again with ethanol 99.5% (Carl Roth GmbH, Germany) for 5min under slow shaking (Microplate mixer MX-M, China). The surfaces were then rinsed in ethanol 99.5% and dried in air. The cleaned coatings were then treated in an in-house designed ultra-violet/ozone (UVO) emitter employing three 4W (12µW/cm^2^ at 1m distance) low pressure mercury vapor lamps (Dinies Technologies GmbH, Villingendorf, Germany). The exposure times were 10min with a 4cm distance between the lamps and the sample’s surface in the same manner as documented. Directly after the UVO treatment, the surfaces were silanized with 3% aminopropyl-triethoxysilane (APTES) in filtered (Rotilabo® syringe filters, CA, pore size 0.20µm, Carl Roth GmbH, Germany) deionized water (ddH_2_O) for 25min at room temperature under slow shaking and washed five times in filtered water. In the next step, the petri dishes were treated with 0.3% glutardialdehyde (GDH) in filtered ddH_2_O for 40min at room temperature under slow shaking and washed again five times in filtered ddH_2_O. A similar treatment with APTES and GDH was previously described.

**Mechanical Characterization**

Cylindrical test samples (diameter 20mm, thickness of 3.5mm) were cut using a purposely-made punch. The mechanical characterization was carried out using two different instruments. A conventional micro-hardness measurement device (FISCHERSCOPE® HM2000, Fischer Instruments, Sindelfingen, Germany) was used to measure the static indentation modulus *E*_IT_ to a depth of approximately 180µm. The test samples were fixed (in-house configuration) on the micro hardness measurement device and the elastomers were applied against the indentation head with a small normal force (< 0.1N). The measurement was performed with the following parameters: force increase maximum test load *F*_IM_ = 16mN, force reduction maximum test load *F*_RM_ = 7.5mN, application time increment *t*_IM_= 20s, application time reduction *t*_RM_= 20s, Mode ${d\sqrt{F}}/{dt}=\mathrm{const}$, indenter type H2N 17201110 Berkovich tip, sample temperature *ϑ* = 20 ± 3°C. The Poisson’s ratio for PDMS samples has been estimated from the literature as *υ* ≈ 0.49. The determination of *E*_IT_ is based on the Oliver-Pharr method. The dynamic storage modulus *G*' and loss modulus G´´ were measured using an Anton Paar (Graz, Austria) rheometer MCR 301 with the following measurement parameter: deformation *γ* = 0.01%, normal force *F*_N_ = 0.1N, angular frequency *ω* = 10s^-1^, sample temperature *ϑ* = 20 ± 2°C (Thermostat Physica 200, measuring head PP20).

**Water Contact Angle Measurement**

The Drop Shape Analyzer (DSA25, Krüss GmbH,Hamburg, Germany) was used in order to determine the water contact angle (CA) of the elastomer formulations. The test samples both with and without APTES and GDH treatment were used for the analysis. The side walls of the petri dishes were removed under filtered (0.2µm) deionized water. The samples were then removed from the water and dried under an air pressure stream before being measured. In the following step a 1.5µl drop of ddH_2_0 was placed on the elastomer surface and the CA of both sides of the drop was measured with the image analysis software (DSA 1.9, Krüss GmbH, Hamburg Germany). Five CA values of one drop were measured and then averaged. The reported CA value was the further average of such measured CA values at 5 spots on different surface areas separated by 3mm. Measurement conditions were as follows: ambient temperature *ϑ* = 23 ± 2°C, relative humidity RH = 36 ± 5%, approximate measurement time per drop *t*_meas_ ≈ 60s.

# Supplementary Figures legends

**Supplementary Figure 1.** **Isolation and Cultivation of murine ON astrocytes.** (**a**) 7 days after the preparation first cells were growing out of the tissue. The retrolaminar ON contains different types of cells. (**b**) oligodendrocytes (**c**) astrocytes and (**d**) microglial cells. (**e,f**) To prove the purity of the astrocyte cell culture, the cells were stained with GFAP, IBA-1, a marker for microglia and with MBP a marker for oligodendrocytes. Neither MBP nor IBA-1 showed any positive staining in the purified c culture (data not shown). Only GFAP (red) showed a positive staining in all cells, indicating the establishment of a pure murine ON astrocytes culture. Nuclei are stained with Dapi (blue).

**Supplementary Figure 2. Elastic properties of PDMS substrates used in this study.** (**a**) Indentation modulus *E*_IT_ of 10, 30 and 60kPa PDMS substrates. (**b**) Complex shear modulus *G* = *G*´ + j*G*´´ of 10, 30 and 60kPa PDMS substrates. (**c**) Water contact angle of all three substrates (10, 30 and 60kPa) is decreased after UVO irradiation and subsequent APTES/GDH treatment.
